# Assessing infection prevention and control programs in residential aged care in Australia: A multi‐methods cross‐sectional study

**DOI:** 10.1111/ggi.14791

**Published:** 2024-01-03

**Authors:** Joanne Tropea, Jill J Francis, Noleen Bennett, Lyn‐li Lim, Deirdre Fetherstonhaugh, Kirsty L Buising, Caroline Marshall, Madelaine Flynn, Wen K Lim, Sanne Peters

**Affiliations:** ^1^ Department of Aged Care Royal Melbourne Hospital Melbourne Victoria Australia; ^2^ Department of Medicine RMH, University of Melbourne Melbourne Victoria Australia; ^3^ Melbourne School of Health Sciences University of Melbourne Melbourne Victoria Australia; ^4^ Department of Health Services Research Peter MacCallum Cancer Center Melbourne Victoria Australia; ^5^ Department of Oncology, Sir Peter MacCallum University of Melbourne Melbourne Victoria Australia; ^6^ Ottawa Hospital Research Institute, Center for Implementation Research Ottawa Ontario Canada; ^7^ Victorian Healthcare Associated Infection Surveillance System (VICNISS) Coordinating Center, Royal Melbourne Hospital Melbourne Victoria Australia; ^8^ National Center for Antimicrobial Stewardship University of Melbourne Melbourne Victoria Australia; ^9^ Department of Nursing, Melbourne School of Health Sciences University of Melbourne Melbourne Victoria Australia; ^10^ Department of Infectious Diseases University of Melbourne at the Peter Doherty Institute for Infection and Immunity Melbourne Victoria Australia; ^11^ Australian Center for Evidence Based Aged Care (ACEBAC) La Trobe University Melbourne Victoria Australia; ^12^ Victorian Infectious Diseases Service, Royal Melbourne Hospital Melbourne Victoria Australia; ^13^ Infection Prevention and Surveillance Service, Royal Melbourne Hospital Melbourne Victoria Australia; ^14^ Infection Prevention, Northern Health Melbourne Victoria Australia; ^15^ Department of Public Health and Primary Care University of Leuven, KU Leuven Louvain Belgium

**Keywords:** infection prevention and control, residential aged care, nursing homes

## Abstract

**Aim:**

To assess infection prevention and control programs in residential aged care facilities.

**Methods:**

A cross‐sectional survey and structured interviews from 10 residential aged care facilities in Victoria, Australia, were used. Infection prevention and control nurse leads from each facility completed a purpose‐built survey based on best practice infection prevention control program core components, including staff training, policies and procedures, governance, and surveillance. Follow‐up interviews with residential aged care staff, residents and family visitors were carried out to elaborate and verify survey data.

**Results:**

Surveys from all 10 facilities were received and 75 interviews carried out. All facilities had an infection prevention and control lead nurse who had undergone additional training, and 60% of facilities had an infection prevention and control lead position description. All facilities had a committee to oversee their infection prevention and control program, and all had policies and procedures for standard and transmission‐based precautions. One facility did not have a policy on healthcare‐associated infection surveillance, and two facilities did not have an antimicrobial stewardship policy. All facilities provided staff training in hand hygiene and personal protective equipment use, but not all routinely assessed competency in these.

**Conclusions:**

The residential aged care facilities' infection prevention and control programs were generally in a strong position, although there were some areas that require improvement. Further assessment of the quality of infection prevention and control program components, such as content of education and training, and policies and procedures, and ongoing evaluation of programs is recommended. **Geriatr Gerontol Int 2024; 24: 358–363**.

## Graphical abstract

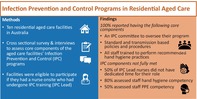

## Introduction

Older people living in residential aged care facilities (RACFs) are susceptible to transmissible organisms, such as influenza, coronavirus disease 2019 (COVID‐19) and norovirus. Infection prevention and control (IPC) programs in RACFs are therefore essential to minimize the risk of infections, and thereby safeguard the health and well‐being of residents. The COVID‐19 pandemic from 2020 disproportionately affected older people living in RACFs in Australia, and investigations highlighted variation in IPC practice and approaches, including the organizational systems, structures and resources, that enable best practice IPC.^1^, ^2^

The World Health Organization (WHO) and the Centers for Disease Control and Prevention provide guidance on IPC program components that should be included in healthcare settings, including RACFs.^3^, ^4^ Although specific recommendations can vary slightly between settings, there is significant overlap in their core IPC program components, which include: (i) staffing and resources, including dedicated IPC trained staff; (ii) policies and procedures; (iii) education and training; (iv) surveillance and monitoring; (v) performance measurement; (vi) implementation of standard and transmission‐based precautions; (vii) outbreak preparedness and responses; and (viii) antimicrobial stewardship (AMS). These core components work together to create a comprehensive IPC program, tailored to the specific needs of each setting.

Another important component of IPC programs is involvement of residents and their families in IPC. The Australian IPC Guidelines recommend organizations provide IPC information to patients and visitors.^5^ They suggest the use of written materials to reinforce verbal discussions as part of their care. Investigations into COVID‐19 outbreaks in Australian RACFs found communication between the RACF and residents and their representatives needed to be strengthened, and providers that had prepared communications strategies were able to better manage the communication needs, and act quickly and proactively during an outbreak.^1^

The WHO cycle to IPC improvement includes baseline assessment of the IPC core components and minimum requirements.^6^ However, little is known about how well IPC programs in RACFs currently align with the recommended components. The present study, therefore, aimed to assess IPC programs in Australian RACFs; and was part of the IMpleMenting Effective infection prevention and control in ReSidential aged carE (IMMERSE) project.^7^

## Methods

### Study design

The present study was a multi‐methods cross‐sectional study using survey and interview data. The findings contributed to the baseline assessment of readiness for IPC practice change that was part of IMMERSE – a larger implementation science project on IPC in residential aged care. The IMMERSE study protocol has been published elsewhere.^7^ The Melbourne Health Human Research Ethics Committee (HREC/81901/MH‐2022) approved the conduct of the study.

### Setting

Participating sites were 10 RACFs in Victoria, Australia. Victoria is the second most populous state in Australia and has >58 000 residential aged care beds provided by private, not‐for‐profit and government organizations.^8^ Facilities were eligible to participate if they were in metropolitan Melbourne and regional Victoria, and had a nurse onsite who had completed or commenced IPC training (IPC lead).^9^ In July 2021, the Australian government introduced the role of IPC leads, and mandated all RACFs have an ongoing dedicated IPC lead nurse on site.

Invitations to participate in IMMERSE were sent to RACFs recommended by IMMERSE investigators. Purposive sampling was used to ensure diversity, and a sample size of 10 facilities was selected based on what was feasible within the scope of the study. A total of 10 RACFs were recruited, with representation of metropolitan and regional locations; small, medium and large sized; private, public and not‐for‐profit providers; and culturally and linguistically diverse RACFs. Collaborative research agreements between the RACF providers and the lead investigator were documented and signed.

### Participants

#### RACF staff

RACF staff were invited to participate in the study, including facility and clinical managers, IPC leads, nurses, personal carers, lifestyle, food and cleaning staff.

IPC leads were informed of the project during the recruitment phase. Informed consent was sought via email and follow‐up phone call. The researchers asked IPC leads and managers to identify staff who would be available to participate in interviews during the site visits. Staff were approached and invited to participate. If they agreed, informed consent was obtained.

#### Residents and family visitors

The researchers asked IPC leads and managers to identify residents and family members who would be available to participate in interviews during the site visits. Residents and family visitors who could understand English, and residents with the capacity to give informed consent were invited to participate in the study. Informed consent was sought before interviews. The participating residents and family members were offered a gift voucher as acknowledgement of the time taken to participate.

### Data collection and analysis

A survey instrument (see File S1) was developed based on the WHO IPC program recommendations, Centers for Disease Control and Prevention Infection Control Assessment and Response (ICAR) Tool for long‐term care, Australian IPC guidelines^5^ and input from IPC experts on the IMMERSE research team. Survey items about the facility and IPC lead; facility‐level IPC guidelines for standard and transmission‐based precautions, and AMS; staff education and training in hand hygiene (HH); and personal protective equipment (PPE) use; infection surveillance; AMS surveillance; and IPC communication with staff, residents and visitors were included.

The survey was pilot tested with three staff from non‐participating facilities using think‐aloud interviews to assess whether items were clear and elicited expected responses.^10^ The think‐aloud method involved asking participants to verbalize their thoughts while completing the survey.^10^ The three staff took 12–15 min to complete the IPC program survey items. Minor changes were made to survey items based on this process, including removal of items that were deemed irrelevant. The final survey consisted of two sections with checkbox and free‐text items: Section 1 – Some information about you and your facility (10 items); and Section 2 – IPC program (39 items). Section 2 asked participants to select from “Yes”/“No”/“Unsure.”

Survey data were collected and managed using the Research Electronic Data Capture tool (REDCap) hosted at the University of Melbourne.^11^ Surveys were sent to the IPC leads, with up to three email reminders. Follow‐up phone calls were made to remind IPC leads who had not completed the survey to do so. Researchers asked IPC leads to complete the surveys before the site visit. At one RACF, the survey was completed during the site visit.

Follow‐up site visits were carried out at each RACF to explore their IPC program in greater depth and verify the survey responses. The researchers used a situated “walk‐and‐talk” approach for interviews with IPC leads to verify locations of IPC‐related procedures, staff training logs, equipment and resources. The “walk‐and‐talk” approach took place within the RACF, and it functioned as a situated interview in which contextual triggers might enhance the participants' descriptions, and make the responses to the interview questions more concrete and locally relevant. Structured interviews were carried out in‐person with staff, residents and family visitors. If family were not present at the RACF, phone interviews were carried out. The interview guides were developed by the research team and refined after the first site visit (See File S2). Researchers conducting the interviews noted down participants' responses as the interviews were not recorded.

Data from the surveys were summarized using descriptive statistics. Interview data were summarized descriptively under each of the IPC program components, and used to verify and elaborate on the survey findings. Where there were discrepancies between survey and interview data, these were discussed with IPC leads and or RACF managers.

Findings from the surveys and interviews were fedback to each facility. A summary of the study findings was presented to participating facilities in the form of a short report (see File S3) and online presentation with discussion, using audit‐and‐feedback principles.^12^, ^13^, ^14^ Specifically, we delivered the feedback in verbal and written (multimodal) formats, co‐presented with a content expert, included explicit targets and an action plan, and the patient/resident voice was incorporated. IPC leads and managers were invited to attend the online discussion that was hosted by a researcher and an IPC expert from the IMMERSE team. Current practices (based on survey and interview data) were compared with guideline recommendations. Where areas for improvement were identified, best‐practice solutions were suggested. Where there was little or no evidence base to support the suggested solutions, the researchers in collaboration with RACF staff determined solutions and made recommendations to address them. It was surmised that these recommendations might leverage simple and existing solutions. After these discussions, RACFs were provided with an action plan template (see File S4) and encouraged to develop 1–2 IPC goals to work towards over the following 12 months.

## Results

The characteristics of the 10 RACFs that participated in the study are described in Table 1. Two were public sector facilities (owned and run by the Victorian Department of Health), four were not‐for‐profit and four were for‐profit facilities. Nine facilities were located in metropolitan Victoria, and one in regional Victoria. The number of residents ranged from 22 to 144, and the physical layout of the RACFs were two (*n* = 5, 50%), three (*n* = 3, 30%) or four (*n* = 2, 20%) wings/houses or ward areas. A total of 60% of the RACFs had all single bedrooms, and 40% had a mix of single and shared bedrooms. Half of the RACFs had all single bathrooms, and the other half had a mix of single and shared bathrooms. The number of staff employed at the RACFs ranged 31–164.

**Table 1:** Characteristics of the residential aged care facilities (*n* = 10)

	Residential aged care facility site									
Characteristic	1	2	3	4	5	6	7	8	9	10
Provider type	Public	NFP	Public	NFP	NFP	For profit	For profit	For profit	NFP	For profit
Location	Metro	Metro	Regional	Metro	Metro	Metro	Metro	Metro	Metro	Metro
No. residents	22	102	24	115	60	99	57	65	52	144
Layout – no. wings/units	2	4	2	4	2	3	2	2	3	3
Room types^†^	Mix	Mix	Mix	All single	All single	All single	All single	All single	All single	Mix
Bathrooms	All shared	Mix	Mix	All single	Mix	All single	All single	All single	All single	Mix
Total no. staff^‡^	31	106	46	164	51	154	57	92	65	

Metro, metropolitan; NFP not for profit.

^†^ Mix of single and shared.

^‡^ Includes casual staff.

A total of 51 staff, 14 residents and 10 family visitors participated in the interviews. The IPC lead from each of the 10 facilities completed the online survey. “Unsure” item responses were followed up by interview during the site visits. Data collection occurred between July and December 2022.

Table 2 presents a summary of IPC program core components.

**Table 2:** Summary of infection prevention and control core components (*n* = 10)

IPC core component	Yes, *n* (%)	Partial, *n* (%)	No, *n* (%)
Staffing and resources			
IPC lead	10 (100)	‐	‐
Dedicated time	5 (50)	‐	5 (50)
IPC lead position description	6 (60)	‐	4 (40)
Policies and procedures			
Standard precautions	10 (100)	‐	‐
Transmission‐based precautions	10 (100)	‐	‐
Infection surveillance	9 (90)	‐	1 (10)
Antimicrobial stewardship	8 (80)	‐	2 (20)
Staff education and training			
Hand hygiene training	10 (100)	‐	‐
Hand hygiene competency assessment	8 (80)	2 (20)	‐
PPE use training	7 (70)	3 (30)	‐
PPE use competency assessment	5 (50)	5 (50)	‐
Mask fit testing	5 (50)	2 (20)	3 (30)
Surveillance and monitoring			
Surveillance programs	10 (100)	‐	
Report up to executive/board	10 (100)	‐	
Report available to care staff	1 (10)	‐	9 (90)
Involving residents and carers			
Staff remind/encourage	7 (70)	3 (30)	‐
IPC information communicated using multiple methods	10 (100)	‐	‐

IPC, infection prevention and control; PPE, personal protective equipment.

### Staffing and resources

All facilities had an IPC lead who had undergone, or was in the process of, completing, additional IPC training. At seven (70%) RACFs, IPC leads had senior/leadership nursing roles, one RACF had a registered nurse in the role and two RACFs had enrolled nurses in the role.

Six (60%) RACFs reported having an IPC lead position description. Five (50%) IPC leads had dedicated time for the role, and 50% had no dedicated time for the role. Three of the IPC leads with dedicated time had ≤1 h per week dedicated time to fulfill IPC role duties (for example, to complete an IPC checklist). All reported feeling supported by their managers, and 40% reported they had access to IPC experts if needed within their organization.

### IPC policies and procedures

All 10 RACFs reported having policies and procedure documents for standard precautions and transmission‐based precautions. Nine (90%) had documents for infection surveillance procedures, and eight (80%) had AMS procedures. All staff interviewed knew how to access their RACF's IPC policies and procedures.

### Staff IPC education and training

The 10 RACFs reported all staff received training in HH. A total of 80% of RACFs reported they assessed HH competency of all staff, and 20% reported assessing most (but not all) staff for HH competency. A total of 50% of RACFs reported assessing all staff, and 50% reported assessing most (but not all) staff in PPE donning and doffing competency. Mask fit testing had not been undertaken at three (30%) RACFs.

### Surveillance and monitoring

All RACFs had surveillance programs for monitoring infections and antimicrobial use. All had reporting mechanisms for surveillance data, most of which reported to a clinical governance or quality committee and then up to the Board. Three RACFs included pharmacists in antimicrobial surveillance. One RACF reported that the pharmacy they used sent doctors regular reports on antimicrobial use, one had a pharmacist leading the AMS reporting, and another had a pharmacist on the advisory committee for AMS.

Only one RACF reported they displayed infection surveillance reports on a staff noticeboard; and one had a staff member (other than the IPC lead or lead nurse) who held the AMS portfolio.

### Involving residents and families in IPC

All RACFs provided written and/or verbal IPC information to residents and families. Residents and family visitors from 30% of RACFs reported they were not always actively assisted with IPC, such as with HH or donning masks. Residents and family visitors from all 10 facilities reported they received IPC information, and this was communicated using multiple methods, such as via newsletters, emails and in person.

### Summary reports

All 10 RACFs were sent the summary report and participated in follow‐up discussions. All RACFs drafted an action plan in response to the feedback, some were developed with the assistance of the research team. Six facilities finalized and signed off on their action plans.

## Discussion

The present study found the IPC programs of all 10 RACFs included the following components: staff with IPC knowledge, IPC policies and procedures, IPC education and training, surveillance and monitoring of infections and antimicrobial use, and IPC communication and involvement with residents and families. Like other studies, we found that not all IPC components were fully met.^15^, ^16^ Most, but not all, RACFs had an AMS procedure in place, and there was variability in assessment of all staff HH competency, staff competency in donning and doffing PPE, and mask fit testing.

The role and duties of the IPC lead varied between sites, and not all had a position description or dedicated time to carry out IPC duties. Given the IPC lead position is a relatively new one, having a written position description would provide guidance to IPC leads regarding their tasks and responsibilities, and the potential to motivate performance.

IPC leads from four RACFs had access to IPC experts within their organization. These were RACFs that were part of a public hospital service or within a large aged care provider. IPC leads need support in having ready access to up‐to‐date, credible information relevant to mandates and recommendations. They often require support in interpreting IPC guidelines and how recommendations can be applied to their setting. This was especially true during the COVID‐19 pandemic; for example, some needed guidance on how to apply zoning with mixed rooms and bathrooms. A community of practice for IPC leads has the potential to provide peer support, share learnings and offer continuing education opportunities for IPC leads.^17^

Sharing of infection data and antimicrobial surveillance data could be improved. Data were generally reported to the quality or clinical governance committee and executive or the Board. Only one facility shared surveillance data with care staff (via staff noticeboard), and none of the facilities routinely shared data with residents or family visitors. Sharing data about antimicrobial use in combination with information about the potential adverse consequences of inappropriate antimicrobial use is an important AMS activity.^18^

In their systematic review, Lee *et al*. examined the effectiveness of IPC interventions in long‐term care and assessed for WHO IPC program components.^19^ Interventions included the components of education and training, monitoring and feedback, surveillance, multimodal strategies, and built environment, materials and equipment. Most (70%) included a combination of components, but none of the studies included all components. There was some evidence to suggest interventions that included education, monitoring and feedback, and four or more multi‐components reduced rates of healthcare‐associated infections. The WHO guidelines on core components of IPC programs^4^ are evidence‐based, but draw largely on research from outside residential aged care, such as from hospital and community studies. The review's findings build on this, and support the evidence base for IPC programs within the context of residential aged care.

To our knowledge, this is the first study to assess core components of IPC programs in Australian RACFs since the COVID‐19 pandemic. We used multiple methods to collect data, and verified survey findings with follow‐up interviews. We included a range of participants in the interviews, including staff from diverse roles, residents and family visitors, to ensure representation from varying perspectives.

The findings were reported back to RACFs using evidence‐based principles to trigger change.^12^, ^14^ Although it is anticipated that evidence‐informed feedback can facilitate practice change, future research should explore the impact of the action plans. Given that staff working in RACFs are time poor, our feedback reports aimed to be brief. Nevertheless, a co‐design process with staff could have informed a more locally appropriate format of feedback, as well as given us a better understanding of the need for feedback. Feedback is commonly provided after an (objective) audit. The present study included more subjective data (surveys and interviews). Future research could explore whether the data collection methods and data sources influence the acceptance of feedback.

There were some limitations to the study design. Most facilities were in the north‐west corridor of metropolitan Melbourne, so might not be representative of other parts of the state or country. We attempted to ameliorate this by sampling facilities from public, private and not‐for‐profit sectors. We collected information about the availability of IPC documents (such as policies and procedures) and IPC‐related activities (such as staff training and education, surveillance), but did not assess or compare the quality of their content. Documentation does not always translate to practice, and we did not assess knowledge of protocols or policies among staff. The results were cross‐sectional rather than longitudinal, and there is recognition that staff turnover is high in the residential care sector. Interviews were brief, not audio‐recorded and summarized descriptively. However, researchers took notes during the interviews, which allowed the team to add to and verify survey findings. The findings provide a valuable snapshot of IPC programs in Australian RACFs. We recognize IPC improvement is a continuous process that requires an enabling environment and multimodal improvement strategies.^6^

The present study showed that the IPC programs in RACFs were generally robust, although there was some room for improvement. Action plans were developed in response to identified gaps, and in accordance with the WHO cycle to IPC improvement. The next steps would be to execute the action plan and evaluate its impact, followed by sustaining the program over the long term. Further investigation of the quality of policies and procedures, and training content is recommended to ensure they align with IPC guidelines.

## Funding information

The IMMERSE study is funded by the Australian Medical Research Future Fund (MRFF) Dementia, Ageing and Aged Care Mission 2020 Grant Opportunity 2021MRF/2007632.

## Disclosure statement

Joanne Tropea and Sanne Peters receive salary support from the MRFF Dementia, Ageing and Aged Care Mission 2020 Grant Opportunity 2021MRF/2007632. All other authors declare no conflict of interest.

## Supporting information


**File S1.** Infection prevention and control program survey.

**File S2.** Interview guide and data collection sheet.

**File S3.** Summary and feedback template.

**File S4.** Action plan template.

## Data Availability

Data available on request from the authors.
