# Supplementary material for: Assessing infection prevention and control programs in residential aged care in Australia: A multi‐methods cross‐sectional study
Source: Geriatr Gerontol Int. 2024 Jan 3;24(Suppl 1):358–63. doi: 10.1111/ggi.14791 (PMC11503640; doi:10.1111/ggi.14791)
Supplement: Supplementary file 1 — File S1. Infection prevention and control program survey. [file GGI-24-358-s003.pdf]

ACTION PLAN
FOR INFECTION PREVENTION AND CONTROL

Goal 1:

Steps to achieve above goal	Who is responsible	Resources	Timeframe	Date achieved	Notes
1.					
2.					
3.					
4.					

Goal 2:

Steps to achieve above goal	Who is responsible	Resources	Timeframe	Date achieved	Notes
1.					
2.					
3.					
4.					

Signature: Date:

Please print name:

Example Goal 1: To develop a position description for the IPC lead role

Steps to achieve above goal	Who is responsible	Resources	Timeframe	Date achieved	Notes
1. Draft position description – include job title, purpose of role, dedicated time, duties, and responsibilities, who role reports to, qualifications	Facility manager<br>IPC lead	No additional resources required	4 weeks		Use organisation's position description template
2. Reviewed by Human resources	Facility manager<br>Human resources		2 weeks		
3. Finalise position description	Facility manager		2 weeks		
4. Get approval from Board/Executive	Facility manager<br>Board/Executive		4 weeks		Dependent on timing of next Board meeting
