# Supplementary material for: Assessing infection prevention and control programs in residential aged care in Australia: A multi‐methods cross‐sectional study
Source: Geriatr Gerontol Int. 2024 Jan 3;24(Suppl 1):358–63. doi: 10.1111/ggi.14791 (PMC11503640; doi:10.1111/ggi.14791)
Supplement: Supplementary file 2 — File S2. Interview guide and data collection sheet. [file GGI-24-358-s002.pdf]

IPC Program assessment – interview guide

IPC lead data collection: Updated to walk and talk with staff. Researcher to take notes.

- Introduction of researcher
- Description of project
- Check we have participant informed consent
- Ask the participant to introduce themselves (including position/profession, years of working experience, numbers of years at that facility, gender, CALD)

IPC lead ☐Consent Manager ☐Consent / NA

QUESTIONS FOR IPC LEAD and/or GENERAL MANAGER

1. Ask IPC lead/s to describe their position at RACF, total FTE, dedicated FTE to IPC role, how long in the role, check details of formal training.

Is there a documented IPC lead Position description? Y/N

2. How are you supported in the role?

What IPC experts are you connected to – describe, how often you communicate with them

3. Ask for details of IPC program review processes – committee, membership, meetings (frequency, content), communication strategies, formal mechanism to include consumer experience and feedback

4. **IPC key performance indicators** – check and follow up on details, what, how, reporting and reviewing processes; other measures such as process and outcome measures, how it is communicated with the team. If no KPIs in IPC – ask about **surveillance of IDs**

IPC Program assessment – interview guide

Policies and Procedures

Check policies and procedures – location, access, get copies

5. Standard precautions Sighted☐ Ask for copy☐
6. Transmission based precautions Sighted☐ Ask for copy☐
7. Staff and resident health and safety Sighted☐ Ask for copy☐
8. Surveillance (if Yes) Sighted☐ Ask for copy☐
9. Antimicrobial Stewardship Sighted☐ Ask for copy☐

10. Who is responsible for influenza and other recommended vaccinations – for staff?

11. Who is responsible for influenza and other recommended vaccinations – for residents?

12. How are policies and procedures communicated with the team?

13. How is IPC staff training monitored? Who provides the staff IPC training? *How is it delivered? How often?*

14. How is IPC information shared with staff – RNs, ENs, cleaning, food services, others?

15. How is IPC information shared with residents? Ask to see flyers/brochures etc.

16. How is IPC information shared with families?

17. Range of masks available Y/N

IPC Program assessment – interview guide

STAFF data collection: Researcher to take notes.

- Introduction of researcher
- Provide description of project, use participant information sheet as guide and get consent.
- Make copy of consent form and give this to participant. If unable to make copy ask for email or for them to take photo.
- Ask the participant to introduce themselves (including position/profession, years of working experience, numbers of years at that facility, gender, CALD)

Staff1 ☐Consent Describe:

Staff2 ☐Consent Describe:

Staff3 ☐Consent Describe:

Staff4 ☐Consent Describe:

Staff5 ☐Consent Describe:

QUESTIONS FOR OTHER STAFF - include ID number next to response

I am going to ask you some questions about infection prevention and control. This includes things you do for COVID or gastro like hand hygiene and wearing a face mask.

1. How is information about infection prevention and control shared with you?

And with other staff – nurses/ cleaning, food services, laundry

2. How are you informed about policies and procedures? (if different from above)

3. How is IPC information shared with residents and family members?
   - How and **who** does this?
   - Ask about their own role

4. What IPC information is included in staff induction/orientation? (Ask new staff members)

5. Have you been trained in Hand Hygiene? How was training delivered? (online, paper based, face to face) who delivered it, when, and how often?

b. Was your hand hygiene knowledge assessed (how – eg paper based, online system)

c. Did someone observe you perform HH (how, who)

6. Have you been trained in PPE use? How was training delivered? (online, paper based, face to face) who delivered it, when, and how often?

b. Was your PPE knowledge assessed (how – eg paper based, online system)

c. Did someone observe you don/doff PPE (how, who)

7. Mask fit testing - have you been fitted Y/N

IPC Program assessment – interview guide

For residents and family interviews

- Introduction of researcher
- Provide description of project, use participant information sheet as guide and get consent.
- Make copy of consent form and give this to participant. If unable to make copy ask for email to send copy to.
- Ask the participant to introduce themselves (resident or family of resident, numbers of years at that facility, relationship to resident, gender, CALD)

Res1 ☐Consent Describe:

Res2 ☐Consent Describe:

Fam1 ☐Consent Describe:

QUESTIONS FOR RESIDENTS and FAMILIES - include ID number next to response

I am going to ask you some questions about infection prevention and control. This includes things you do for COVID or gastro like hand hygiene and wearing a face mask.

1. Resident and family –are you aware of rules about hand hygiene, wearing a mask and other things related to keeping infections out of this facility.

b. Do you feel you receive enough information from the facility about ways you can help prevent infections? If no what else would you like to know?

c. How do you receive this information – written, email, in person. How do or would you like to receive information about IPC?

d. How are you encouraged by staff here to participate in IPC?

2. Has someone from this facility explained cough etiquette to you? Hand hygiene? Wearing a mask?

b. Who provided this information and how was this information provided?

3. Other – has the facility/staff here informed you about infection numbers (b. or appropriate and inappropriate use of antibiotics)
