# Supplementary material for: Assessing infection prevention and control programs in residential aged care in Australia: A multi‐methods cross‐sectional study
Source: Geriatr Gerontol Int. 2024 Jan 3;24(Suppl 1):358–63. doi: 10.1111/ggi.14791 (PMC11503640; doi:10.1111/ggi.14791)
Supplement: Supplementary file 3 — File S3. Summary and feedback template. [file GGI-24-358-s001.pdf]

# SURVEY FOR THE INFECTION PREVENTION AND CONTROL (IPC) LEAD

Survey instructions

Thank you for agreeing to complete this survey. The survey asks questions about the infection prevention and control (IPC) program at your facility. It will take 20-30 minutes to complete.

Please note this is not a test. You may require some help from your manager or other staff to answer some of the questions. And you can look up IPC policies and procedures if needed.

If you are unsure about any questions or would like more information, please contact "INSERT Name of Reseacher and contact details".

## Section 1: Some information about you and ADD name of FACILITY

First name

__________________________________

Last name

__________________________________

Please select your position/role (you may select more than one)

- ☐ Infection prevention and control (IPC) lead
- ☐ General manager
- ☐ Clinical care coordinator
- ☐ Nurse unit manager
- ☐ Care manager
- ☐ Other

Other - Please describe:

__________________________________

Years you have worked in residential aged care

- ◯ Less than 5 years
- ◯ 5-10 years
- ◯ More than 10 years

Years you have worked at (NAME OF RACF)

- ◯ Less than 5 years
- ◯ 5-10 years
- ◯ More than 10 years

## Section 1: Some information about you and ADD name of FACILITY

Number of residents

__________________________________

Layout of residents' rooms at your facility

- ◯ All single bed rooms
- ◯ Mix of single bed and shared rooms
- ◯ All shared rooms

Bathrooms at your facility

- ◯ All individual bathrooms
- ◯ All shared bathrooms
- ◯ Mix of individual and shared bathrooms

Number of outbreaks over the past 12 months

__________________________________

List the infection type and date for each outbreak.
Dates can be approximate

__________________________________

## Section 2: Infection prevention and control (IPC) program

IPC lead in residential aged care

| | |
|---|---|
| A. The facility has a specified person/s onsite who is responsible for coordinating the IPC program | ◯ Yes<br>◯ No |
| B. The person/s responsible for coordinating the IPC program has commenced or completed formal training in IPC | ◯ Yes<br>◯ No |
| If YES, please select the course | ◯ Foundations of IPC for Aged Care Staff, ACIPC<br>◯ Graduate Certificate in IPC, Griffith University<br>◯ Master in IPC, Griffith University<br>◯ Graduate Certificate in Infection Control, James Cook University<br>◯ Graduate Certificate in Nursing Science (Infection Control Nursing), University of Adelaide<br>◯ Other |
| Other - Please describe: | __________________________________ |

## Standard precautions

**C. The documented policies and procedures on standard precautions include information about:**

| | Yes | No | Unsure |
|---|---|---|---|
| i. Hand hygiene | ○ | ○ | ○ |
| ii. The use of of appropriate personal protective equipment (PPE) | ○ | ○ | ○ |
| iii. The safe use and disposal of sharps | ○ | ○ | ○ |
| iv. Routine environmental cleaning | ○ | ○ | ○ |
| v. Respiratory hygiene and cough etiquette | ○ | ○ | ○ |
| vi. Aseptic technique | ○ | ○ | ○ |
| vii. Waste management | ○ | ○ | ○ |
| viii. Appropriate handling of linen | ○ | ○ | ○ |

## D. All staff receive training and education (specific to their job role) in:

| | Yes | No | Unsure |
|---|---|---|---|
| i. Standard precautions at time of employment | ◯ | ◯ | ◯ |
| ii. Standard precautions annually | ◯ | ◯ | ◯ |

## E. All nurses and personal carers receive formal training on:

| | Yes | No | Unsure |
|---|---|---|---|
| i. Proper use of PPE at the time of employment | ◯ | ◯ | ◯ |
| ii. Proper use of PPE annually | ◯ | ◯ | ◯ |

## F. All nurses and personal carers have an assessment of their competency of :

| | Yes | No | Unsure |
|---|---|---|---|
| i. Proper use of PPE at the time of employment | ◯ | ◯ | ◯ |
| ii. Proper use of PPE annually | ◯ | ◯ | ◯ |

G. Nurses and personal carers support the residents to practice hand hygiene and respiratory etiquette. ◯ Yes ◯ No ◯ Unsure

H. The facility supports families and visitors to practice standard precautions, such as hand hygiene. ◯ Yes ◯ No ◯ Unsure

If YES, please provide an example: ______________________

**Transmission-based precautions**

**I. The documented policies and procedures on transmission-based precautions include information about:**

| | Yes | No | Unsure |
|---|---|---|---|
| i. Continued implementation of standard precautions | ○ | ○ | ○ |
| ii. Appropriate use of personal protective equipment (including gloves, apron or gowns, surgical masks or P2 respirators, and protective eyewear) | ○ | ○ | ○ |
| iii. Resident-dedicated equipment | ○ | ○ | ○ |
| iv. Allocation of single rooms or cohorting of patients | ○ | ○ | ○ |
| v. Enhanced cleaning and disinfecting of the patient environment | ○ | ○ | ○ |
| vi. Restricted transfer of residents within and between facilities | ○ | ○ | ○ |

## J. All staff receive training and education (specific to their job role) in:

| | Yes | No | Unsure |
|---|---|---|---|
| i. Transmission-based precautions at time of employment | ◯ | ◯ | ◯ |
| ii. Transmission-based precautions annually | ◯ | ◯ | ◯ |

## Management and clinical governance in IPC

## K. The facility has a process for reporting and reviewing:

| | Yes | No | Unsure |
|---|---|---|---|
| i. IPC program, strategies and plans (such as quality or IPC committee) | ◯ | ◯ | ◯ |
| ii. IPC surveillance data (such as quality or IPC committee) | ◯ | ◯ | ◯ |

If YES, please describe: __________________________________

L. IPC key performance indicators (KPIs) are collected and reviewed

◯ Yes
◯ No
◯ Unsure

If YES, please describe __________________________________

M. IPC responsibilities are included in all staff position descriptions. You may need to check with manager

◯ Yes
◯ No
◯ Unsure

## Staff and resident health and safety

| | |
|---|---|
| N. Is there a workplace vaccination program for staff that includes influenza and COVID-19? | ◯ Yes<br>◯ No |
| O. Are there documented policies available for all staff with potentially transmissible conditions (such as leave policy)? | ◯ Yes<br>◯ No<br>◯ Unsure |
| P. Is there a vaccination program for residents that includes influenza and COVID-19? | ◯ Yes<br>◯ No |

## Surveillance

Q. Does your facility have a surveillance system that monitors infections?

◯ Yes
◯ No
◯ Unsure

If YES, please describe

________________________________

R. Is the surveillance system able to detect early outbreaks?

◯ Yes
◯ No
◯ Unsure

## S. Documented policies and procedures are available for:

| | Yes | No | Unsure |
|---|---|---|---|
| i. Identification of potentially infectious residents at time of admission | ○ | ○ | ○ |
| ii. Response to laboratory notification of infectious pathogens | ○ | ○ | ○ |

## Antimicrobial Stewardship (AMS)

| | |
|---|---|
| T. Does the facility have an antimicrobial stewardship (AMS) program? | ◯ Yes<br>◯ No<br>◯ Unsure |
| U. Are there documented policies and procedures for AMS? | ◯ Yes<br>◯ No<br>◯ Unsure |
