# Supplementary material for: Assessing infection prevention and control programs in residential aged care in Australia: A multi‐methods cross‐sectional study
Source: Geriatr Gerontol Int. 2024 Jan 3;24(Suppl 1):358–63. doi: 10.1111/ggi.14791 (PMC11503640; doi:10.1111/ggi.14791)
Supplement: Supplementary file 4 — File S4. Action plan template. [file GGI-24-358-s004.pdf]

IPC PROGRAM COMPONENTS

What we did: We asked the IPC lead to complete an online survey about the IPC program at <Name of RACF>. This included questions about policies and procedures, staff training and infection surveillance.

We visited the aged care home and interviewed four staff, two residents and one family visitor.

Explanation of Results summary table
Column 1: Targeted practice: The red text refers to the questions asked in the IPC lead survey and interviews conducted with the RACF staff.
The italics text presents recommendations taken from the Australian Guidelines for the Prevention and Control of Infection in Healthcare (2019) and the Center for Disease Control Infection Control Assessment and Response (CDC ICAR) tool for nursing homes.

Column 2: Current practice: Presents a summary of the data collected from the IPC lead survey and staff interviews.

Column 3: Suggestions: Presents suggestions from the IMMERSE team based on differences between targeted and current practice.

Column 4: Action Plan: Please review the suggestions and discuss what is feasible and how you would go about achieving the suggestions (action plan). The action plan for improving practice will be documented during the presentation and discussion of Phase 1 IMMERSE project findings.

The Action Plan: The Action Plan will be discussed and agreed by the RACF staff with the support of the IMMERSE research team. It will include a clear goal (what you want to achieve), who will be responsible and involved in achieving the goal, and the anticipated time frame (when you expect to achieve it). The Action Plan needs to be feasible for you and your facility. We will work with you to identify the priority areas and how best we can support you to achieve this.

What we found: See table below.

Targeted practice – what the guidelines recommend	Current practice – what is being done	Suggestions	Action plan (who, what, when)
**STANDARD & TRANSMISSION-BASED PRECAUTIONS**			
We asked:<br>Is there a policy and procedure on standard precautions (hand hygiene, PPE, cleaning, waste management etc)?<br>Do staff know how to access the policy and procedure?<br>*The facility has a policy on Standard Precautions which includes selection and use of PPE (CDC IPCAR).*	You said:		
We asked: Do all staff undertake hand hygiene training at induction?<br>*Job-specific training should be provided as: part of orientation, when new procedures affect the employee's occupational exposure, and before rostering to a hazardous area. Education activities can be integrated into staff orientation programs, credentialing packages, annual training, and competency testing…*			
We asked: Do all staff undertake hand hygiene training regularly?<br>*Healthcare workers competency should be assessed, and records should be maintained of their participation in education programs.*			
We asked: Do all staff undertake hand hygiene competency regularly?			
We asked: Do all staff undertake PPE training regularly?			

Targeted practice – what the guidelines recommend	Current practice – what is being done	Suggestions	Action plan (who, what, when)
We asked: Do all staff undertake PPE competency regularly?			
We asked: Have all staff undergone mask fit testing?<br>*P2 respirator: it is essential that the wearer is properly fitted and trained in its safe use. Healthcare workers are encouraged to actively observe each other's mask fitting and immediately advise of any fitting issues to maximise healthcare worker and patient safety. Healthcare workers must perform fit checks every time they put on a P2 respirator to ensure it is properly applied.*<br>*Fit testing programs may be considered: At commencement of employment; when there is significant change in the wearer's facial characteristics; at regular intervals Standard AS/NZS 1715: 2009 recommends annual fit testing.*			
We asked:<br>Is there is a policy and procedure on transmission-based precautions?<br>Do staff know how to access the policy and procedure?<br>*The facility has a policy on Transmission-based Precautions that includes the clinical conditions for which specific PPE should be used.*<br>*Become familiar with local policy on appropriate PPE, and when it should be*			

Targeted practice – what the guidelines recommend	Current practice – what is being done	Suggestions	Action plan (who, what, when)
put on and taken off, when attending patients on transmission-based precautions (National GLs).<br>*Become familiar with local policy on the implementation of transmission-based precautions in the event of an outbreak (National GLs).*			
**SURVEILLANCE OF INFECTIONS & ANTIMICROBIAL USE**			
We asked:<br>Does the RACF have a surveillance system to monitor infections?<br>Is there an infection surveillance policy and procedure?<br>What are the reporting mechanisms?<br>*The current evidence does not support routine screening for multiresistant organisms (MROs) in residential aged care.*<br>*Monitoring and review is an essential component of the risk-management process.*			
We asked:<br>Is there an AMS program, including surveillance and reporting of antimicrobial use?<br>Is there an AMS policy and procedure?<br>*Antimicrobial stewardship programs should be implemented in residential care. There is a strong need to implement AMS Programs in RACFs where the infection risk for residents is high.*			
**IPC COMMUNICATION**			

Targeted practice – what the guidelines recommend	Current practice – what is being done	Suggestions	Action plan (who, what, when)
We asked:<br>Is IPC information communicated with staff using multiple strategies (for example verbal and written information)?<br>*Communication and consultation are also key elements of clinical risk management. An interactive exchange of information between management, healthcare workers, patients and other stakeholders provides the basis for increased awareness of the importance of infection prevention and control, identification of risks before they arise and prompt management of risks as they occur.*<br>*Outbreak: Within a healthcare facility, effective communication could consist of:*<br>*• appropriate signage to limit access to a room or a clinical unit*<br>*• electronic alerts on the medical record to manage cases and contacts*<br>*• emails and multimedia to target all stakeholders within the healthcare facility*<br>*• provision of education and written materials to visitors to inform them of the situation and the infection control measures with which they should comply.*			
We asked:			

Targeted practice – what the guidelines recommend	Current practice – what is being done	Suggestions	Action plan (who, what, when)
Is IPC information communicated with residents and families using multiple strategies (for example verbal and written information)?<br>*…healthcare workers should ensure that their patients understand why certain practices are being undertaken, and that these practices are in place to protect everyone from infection.*			
**INVOLVING RESIDENTS AND FAMILY IN IPC**			
We asked:<br>Are residents and families encouraged to participate in IPC (educated by staff, supported by RACF to practice IPC)?<br>• *Healthcare facilities need to take an organisational approach to involving patients in their care.*<br>• *A patient-centred health system is known to be associated with safer and higher quality care.*<br>• *A two-way approach that encourages patient participation is essential to successful infection prevention and control.*			
**OTHER - IPC Lead**			
We asked:<br>Is there an IPC lead position description (or similar)?<br>Does the IPC lead have dedicated time or FTE for IPC activities?<br>How is the IPC lead supported?			

Targeted practice – what the guidelines recommend	Current practice – what is being done	Suggestions	Action plan (who, what, when)
There must be adequate resourcing for dedicated infection control staff, and resources to run the IPC program including professional development.			

Abbreviations: AMS antimicrobial stewardship; HH hand hygiene; HS hand sanitise; IPC infection prevention and control; PPE personal protective equipment; RACF residential aged care facility; RAT rapid antigen test

Discussion points
